# 1565. Antiretroviral Stewardship: Protocol Development and Implementation at an Academic Medical Center.

**DOI:** 10.1093/ofid/ofad500.1400

**Published:** 2023-11-27

**Authors:** Tara Bylsma, Katlyn Grossman, Tine Vindenes

**Affiliations:** Tufts Medical Center, West Roxbury, Massachusetts; Tufts Medical Center, West Roxbury, Massachusetts; Tufts Medical Center, West Roxbury, Massachusetts

## Abstract

**Background:**

1.2 million people are living with HIV (PLWH) in the USA. As this population grows older (47% of PLWH were over age 50 in 2018) and more medically complex, the risk of medication errors increases. This population has not been prioritized for stewardship efforts, but experts have called for ARV stewardship programs to be developed. We developed a protocol to systematically review patients on ARVs during inpatient admissions to reduce and prevent medication errors, improve patient safety, and improve equity of care for PLWH.

**Methods:**

We implemented a pharmacist-driven intervention over a 3-month period and compared it to usual practice in the 3 months prior to launch. Patients with active ARV prescriptions were identified using a novel automated patient report generated within the EMR. Then, a pharmacist reviewed the ARV regimen and opportunistic infection prophylaxis for accuracy, completeness, potential DDIs or administration issues. A standardized consult note was developed to communicate pharmacist recommendations to the care team.

We evaluated qualitative features of the ARV-related interventions and calculated the proportion of hospital days with and without medication errors.

**Results:**

The baseline period included 52 patients with an average age of 52 years and LOS of 6.6 days. The intervention period included 60 patients with an average age of 53 years and LOS of 5.4 days. During the intervention, automated pharmacy review led to medication intervention on 49% of patients, compared with 36% of patients in the baseline period. Similarly, the average time to pharmacy intervention decreased from 3 days after admission in the baseline period to 2.5 days in the intervention period.

The most common interventions were resuming an ARV regimen and management of DDIs. The most common clinical rationales for interventions were DDIs, acute changes in renal function, and non-formulary medication restrictions.

Automated patient identification and pharmacist review reduced the prevalence of medication errors related to ART from 17% of inpatient days to 2% of inpatient days.

**Conclusion:**

There is a need for ARV stewardship. Expanding, automating, and standardizing these capabilities improved patient safety by reducing medication errors and improving continuity of care in the inpatient setting.

**Disclosures:**

**All Authors**: No reported disclosures

